# Embryo transfers between C57BL/6J and DBA/2J mice: Examination of a maternal effect on ethanol teratogenesis

**DOI:** 10.3389/fgene.2014.00436

**Published:** 2014-12-11

**Authors:** David Gilliam

**Affiliations:** School of Psychological Sciences, University of Northern ColoradoGreeley, CO, USA

**Keywords:** fetal alcohol spectrum disorders/genetics, mice, inbred strains, teratogenesis, embryo transfer

## Abstract

Genetic factors influence fetal alcohol spectrum disorders (FASDs) in both humans and animals. Experiments using inbred and selectively bred mouse stocks that controlled for (1) ethanol dose, (2) maternal and fetal blood ethanol levels, and (3) fetal developmental exposure stage, show genotype can affect teratogenic outcome. Other experiments distinguish the teratogenic effects mediated by maternal genotype from those mediated by fetal genotype. One technique to distinguish maternal versus fetal genotype effect is to utilize embryo transfers. This study is the first to examine ethanol teratogenesis – fetal weight deficits and mortality, and digit, kidney, and vertebral malformations – in C57BL/6J (B6) and DBA/2J (D2) fetuses that were transferred as blastocysts into B6 and D2 dams. We hypothesized that, following maternal alcohol exposure, B6 and D2 fetuses gestating within B6 mothers, as compared to D2 mothers, will exhibit a higher frequency of malformations. On day 9 of pregnancy, females were intubated (IG) with either 5.8 g/kg ethanol (E) or maltose-dextrin (MD). Other females were mated within strain and treated with either ethanol or maltose, or were not exposed to either treatment. Implantation rates were affected by genotype. Results show more B6 embryos implanted into D2 females than B6 females (*p* < 0.05; 47% vs. 23%, respectively). There was no difference in the percentage of D2 embryos implanting into B6 and D2 females (14 and 16%, respectfully). Litter mortality averaged 24% across all experimental groups. Overall, *in utero* ethanol exposure reduced mean litter weight compared to maltose treatment (*E* = 1.01 g; MD = 1.19 g; *p* < 0.05); but maltose exposed litters with transferred embryos weighed more than similarly treated natural litters (1.30 g vs. 1.11 g; *p* < 0.05). Approximately 50% of all ethanol exposed B6 fetuses exhibited some malformation (digit, vertebral, and/or kidney) regardless of whether they were transferred into a B6 or D2 female, or were naturally conceived. This suggests the D2 maternal uterine environment did not offer any protection against ethanol teratogenesis for B6 fetuses. One of the questions remaining is the how the B6 uterine environment affects D2 teratogenesis. No definitive conclusions can be drawn because too few viable D2 litters were produced.

## INTRODUCTION

Women who drink alcohol while pregnant risk having children with congenital malformations (Jones and Smith, 1973, 1975). In the most extreme cases Fetal Alcohol Syndrome (FAS) may be diagnosed. The syndrome is hallmarked by pre- and postnatal growth retardation, craniofacial abnormalities, and central nervous system dysfunction including behavioral abnormalities. The designation fetal alcohol spectrum disorder (FASD) is now used as an umbrella term covering all outcomes associated with prenatal alcohol exposure. Not all children exposed to alcohol *in utero* display FASD characteristics. This indicates individual differences (both maternal and fetal) in ethanol teratogenesis susceptibility. Many risk factors play a role in FASD development and several studies point to genetic differences in susceptibility.

Mice are useful in studying FASD. All hallmark features of FASD can be replicated in mice. Experiments using inbred and selectively bred mouse stocks show genotype can affect teratogenic outcome (Warren and Li, 2005). The deleterious outcomes under genetic control include differing levels of embryo lethality; brain morphology; fetal weight gain; and digit, skeletal, ocular, renal, and heart anomalies; and behavioral anomalies (Gilliam et al., 1987, 1989; Goodlett et al., 1989; Gilliam and Kotch, 1990, 1992, 1996; Boehm et al., 1997).

Some teratogenic effects are mediated by maternal genotype and may be distinguished from those mediated by fetal genotype (Gilliam and Irtenkauf, 1990; Gilliam et al., 1997, 2011; Downing and Gilliam, 1999). Reciprocal breeding between distinct mouse stocks identify these distinctions. A maternal effect is indicated when genetically identical heterozygous offspring differ in responses based on which homozygous mouse stock is used as the mother in the reciprocal cross (Biddle and Fraser, 1977). If the difference is limited to male offspring or observed at a higher rate in males, the maternal effect may be attributed to X-linked genes. When the difference is not male-specific, maternal effects are presumably due to cytoplasmic inheritance, maternal physiology, or epigenetic phenomena.

When C57BL/6J (B6) mice are crossed with any other mouse stock, having a B6 mother significantly increases malformation frequency compared to not having a B6 mother (Gilliam and Irtenkauf, 1990; Gilliam et al., 1997, 2011; Downing and Gilliam, 1999). In one study (Downing and Gilliam, 1999) we found a maternal genetic effect on vertebral malformations in reciprocal crosses of B6 and DBA/2J (D2) mice following alcohol exposure. Furthermore, the source of the maternal effect could not be ascribed to sex-linked genes or factors transmitted through the egg cytoplasm. Two other factors may account for a maternal effect. They are maternal uterine environment or epigenetic phenomena. In the present study we used embryo transfers to examine the independent effect of maternal uterine environment on ethanol teratogenesis. Genetically identical embryos can be implanted into pseudo-pregnant recipient females with differing genotypes. If treatment effects on these offspring differ, the difference can be attributed to maternal genotype. Conversely, embryos of differing genotype can be implanted into genetically identical recipient females. If treatment effects on these offspring differ, it can be attributed to fetal genotype. Because we previously ruled out sex-linked genes and cytoplasmic factors accounting for the maternal effect in crosses of B6 and D2 mice, we hypothesized that the B6 uterine environment is responsible for increased vertebral malformation in D2 fetuses. This is the first study to examine how maternal uterine environment affects ethanol teratogenesis using embryo transfers.

## MATERIALS AND METHODS

### EXPERIMENTAL ANIMALS AND EMBRYO TRANSFER PROCEDURES

Experimental animals were B6 and D2 mice obtained from the Jackson Laboratory (Bar Harbor, ME, USA) and maintained on a normal light/dark cycle (lights on at 0700 h). All embryo transfers were completed by James Gross, Mouse Genetics Core Facility, National Jewish Health (NJH), 1400 Jackson St., Denver, CO 80206, USA. Seven or eight 3.5-day blastocysts of the same genotype were obtained from approximately 10-week old donor females. These were implanted into each oviduct of pseudo-pregnant recipient 10-week old recipient females (2.5 days *post coitus*). By design, donor and recipient female ages were consistent with those used in our previous studies (Boehm et al., 1997; Downing and Gilliam, 1999). Subjects recovered from surgery at NJH and were transported to the Animal Facility at the University of Northern Colorado (UNCO) on day 7 of pregnancy.

### EXPERIMENTAL TREATMENTS

Between 1230 and 1330 h on day 9 of pregnancy, females were intragastrically intubated with either 5.8 g/kg ethanol (20% w/v) or an isocaloric amount of a maltose-dextrin solution (35% w/v). Naturally conceived (no embryo transplant) fetal groups were established by mating females with same-strain males. These females were treated with either ethanol or maltose-dextrin as described above. Three B6 females mated to a B6 male were inadvertently not intubated. They remained in the experiment as an unexposed control group.

### TERATOLOGICAL ASSESSMENT

On day 18 of pregnancy females were killed by CO_2_ inhalation and necropsied between 1400 and 1700 h. Uterine horns were exposed and a count made of live and resorbed fetuses. Live fetuses were sexed, weighed, and prepared for either skeletal or soft tissue examination, as previously described Gilliam et al. (2011). All live fetuses were examined for digit malformations (ectrodactyly, syndactyly). Approximately half of each litter was examined for vertebral malformations (missing or fused vertebral arches and/or centra), while the remaining half was examined for kidney malformations (hydronephrosis, missing kidney). Examination of uterine horn contents and fetal malformations was conducted by the same person (DG), who was blind to prenatal treatment and transferred embryo genotype.

### PROTOCOL APPROVAL

Experimental procedures were approved by the Institutional Animal Care and Use Committees of the University of Northern Colorado (Protocol 239-01) and National Jewish Health (Protocol AS2788-05-11). All procedures were conducted in accordance with the Guide for the Care and Use of Laboratory Animals [National Research Council (U.S.). Committee for the Update of the Guide for the Care and Use of Laboratory Animals et al., 2011].

### STATISTICAL ANALYSES

Mean litter weight and percent litter mortality were examined by ANOVA with implant status (naturally conceived and transferred), maternal genotype (B6 and D2), fetal genotype (B6 and D2), and prenatal treatment (maltose, ethanol, and non-intubated) as grouping factors. The proportion of successful implantations was calculated. The total number of embryos successfully implanted was divided by the total number transferred. Successful-implantation proportions for the same embryo genotype (either B6 or D2) were compared between maternal genotypes (B6 vs. D2) using the *z*-test for comparing two proportions, as described by Bluman (2012). Since ethanol or maltose-dextrin treatments were given 2–3 days after embryos normally achieve uterine implantation, treatment effects on implantation success were not examined. Comparisons of malformations were conducted using the Fisher ’s Exact Test Calculator for 2 × 2 Contingency Tables at: http://research.microsoft.com/en-us/um/redmond/projects/mscompbio/fisherexacttest/.

## RESULTS

### TRANSFER SUCCESS RATE

A total of 294 B6 blastocysts and 196 D2 blastocysts were transferred to B6 or D2 oviducts (**Table 1**). More B6 embryos successfully implanted into D2 dams than into B6 dams (*z*-test for comparing two proportions = -4.29, *p* < 0.05). In contrast, there were no differences in the proportion of D2 embryos that successfully implanted into D2 or B6 dams (*z* = 0.27; *p* > 0.05). Teratogenic effects are often calculated on a per litter basis, therefore only litters with more than one implantation site are typically included in tallying fetal mortality and/or malformations. **Table 1** includes both the number of successful implants and proportions of litters with more than one successful implant. Verification of zero implantations was made by compressing uterine horns between two microscope slides.

**Table 1:** Embryo transfer and implantation success rates.

	B6 Dams^a^				D2 Dams^b^			
	Total blastocysts transferred	Successful implants	Total litters	Total litters with > 1 implant	Total blastocysts transferred	Successful implants	Total litters	Total litters with > 1 implant
B6 Embryos	150	35 (23.3%)	10*	6	144	68 (47.2%)	9	7
D2 Embryos	126	18 (14.3%)	9	2	70	11 (15.7%)	5*	3

*^a^Three B6 dams had zero B6 implants and seven had zero D2 implants*.

*^b^Two D2 dams had zero B6 implants and one had zero D2 implants*.

*One B6 litter had only one successful B6 embryo implantation and one D2 litter had only one successful D2 embryo implantation.

### LITTER MORTALITY

Litter mortality data were calculated by tabulating early and late resorptions, as differentiated by Gleich and Frohberg (1977), and dividing by total implantations. Average percent litter mortality for each treatment and genotype combination is shown in **Table 2**. Mortality tended to be higher for litters with transferred embryos (34%) than for naturally conceived litters (15%). However, analysis (ANOVA) of litter mortality indicated no effects of pregnancy type (transferred vs. natural), maternal genotype, fetal genotype, or treatment, nor were there any interactions.

**Table 2:** Mean percent litter mortality (±SEM).

	B6 Transferred		D2 Transferred		B6 Natural			D2 Natural	
	Maltose (*n* = 3)	Ethanol (*n* = 4)	Maltose	Ethanol (*n* = 2)	Unexposed (*n* = 3)	Maltose (*n* = 7)	Ethanol (*n* = 8)	Maltose (*n* = 2)	Ethanol (*n* = 3)
B6 Dam	33.3 (16.7)	39.4 (16.3)	NA	44.4 (0)	11.1 (11.1)	20.2 (8.2)	10.1 (3.9)	22.7 (22.7)	14.3 (14.3)
	
	**Maltose(*n* = 2)**	**Ethanol(*n* = 5)**	**Maltose(*n* = 2)**	**Ethanol(*n* = 2)**					
	
D2 Dam	43.7 (6.2)	44.9 (14.5)	0	12.5 (12.5)

*NA – data not available. Of the 9 B6 dams implanted with D2 embryos, three were treated with maltose, but none had any implants or live pups*.

### LITTER WEIGHT

A total of 43 litters had one or more live pups (see **Table 3** for n’s). Each pup was weighed on an electronic balance to 0.001 g. Treatment effects on average litter weight for transferred embryos and naturally conceived litters (within strain mating) for each genotype combination are shown in **Table 3**.

**Table 3:** Mean litter weight in grams (±SEM).

	B6 Transferred		D2 Transferred		B6 Natural			D2 Natural	
	Maltose (*n* = 3)	Ethanol (*n* = 4*)	Maltose	Ethanol (*n* = 2)	Unexposed (*n* = 3)	Maltose (*n* = 7)	Ethanol (*n* = 8)	Maltose (*n* = 2)	Ethanol (*n* = 3)
B6 Dam	1.333 (0.098)	1.064 (0.085)	NA	0.830 (0.078)	1.205 (0.027)	1.171 (0.039)	1.064 (0.037)	0.884 (0.021)	0.984 (0.090)
	
	**Maltose(*n* = 2)**	Ethanol(*n* = 5*)	**Maltose(*n* = 2)**	**Ethanol(*n* = 2)**					
	
D2 Dam	1.292 (0.001)	0.954 (0.052)	1.267 (0.060)	1.061 (0.270)					

NA – data not available. Of the 9 B6 dams implanted with D2 embryos, three were treated with maltose, but none had any implants or live pups.

*Includes one litter with more than one implant but only one live pup.

**FIGURE 1:**
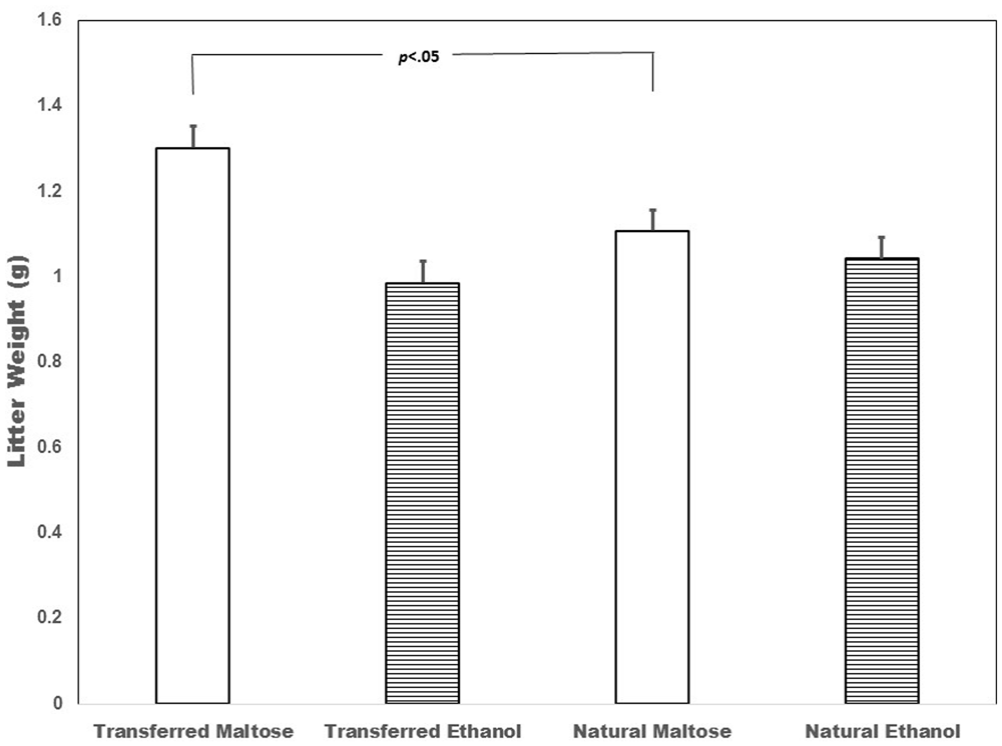
**Fetal weight (grams) was similarly affected by ethanol treatment in both transferred and naturally conceived fetuses (*p* > 0.05), while maltose-exposed fetuses resulting from embryo transfers weighed more than similarly treated naturally conceived fetuses (*p* < 0.05)**.

Analysis of mean litter weight showed a main effect of treatment [ethanol < maltose; *F*(2,42) = 4.58, *p* < 0.05] and an interaction between pregnancy type (transferred vs. natural) and treatment [ethanol vs. maltose; *F*(1,42) = 4.48, *p* < 0.05]. Litter weight for both transferred and natural litters was similarly decreased by ethanol exposure. In contrast, maltose exposed litters with transferred embryos weighed more than similarly treated natural litters (*p* < 0.05; **Figure 1**).

### FETAL MALFORMATIONS

Malformations were tabulated for digits, vertebra, and kidneys and are shown in **Table 4**. As expected, ethanol exposed B6 fetuses exhibited more malformations than ethanol exposed D2 fetuses (Fisher’s exact test, *p* = 0.06) regardless of implantation status (naturally conceived or transferred). In fact, approximately 50% of all ethanol exposed B6 fetuses had some malformation. Importantly, malformation frequency for B6 fetuses did not depend on whether they were naturally conceived or transferred into D2 or B6 dams. This suggests the D2 uterine environment does not provide B6 fetuses protection from *in utero* alcohol exposure, meaning no maternal genotype effect. In contrast to B6 fetuses, ethanol exposed D2 fetuses showed very few malformations. The one exception was ethanol exposed D2 fetuses transferred to B6 dams; 50% had some malformation. In particular, *in utero* ethanol increased vertebral malformations from 0% in D2 fetuses transferred to D2 dams to 67% in D2 fetuses transferred to B6 dams. This suggests the B6 uterine environment may increase malformation frequency in D2 fetuses, a maternal genotype effect. However, given the low numbers of ethanol exposed D2 embryos, malformation frequency was not significantly different from other ethanol exposed D2 groups.

**Table 4:** Digit, kidney, and vertebral malformations.

		B6 Transferred		D2 Transferred		B6 Natural			D2 Natural	
		Maltose	Ethanol	Maltose	Ethanol	Unexposed	Maltose	Ethanol	Maltose	Ethanol
B6 Dam	Digit	0/6	9/18	NA	0/10	0/25	0/42	20/69	0/11	0/16
	Kidney	0/1	1/5	NA	1/4	0/13	2/24	9/38	1/5	2/9
	Vertebral	0/5	2/13	NA	4/6	0/12	0/28	7/31	1/6	0/7
	ANY	0/6	9/18		5/10	0/25	2/42	30/69	2/11	2/16
D2 Dam	Digit	0/7	10/33	0/6	0/4					
	Kidney	0/3	5/15	0/3	1/1					
	Vertebral	0/4	5/18	0/3	0/3					
	ANY	0/7	16/33	0/6	1/4					

*Table numbers represent number of fetuses with a malformation (numerator) divided by total number of fetuses examined (denominator).*

*NA – data not available. Of the 9 B6 dams implanted with D2 embryos, three were treated with maltose, but none had any implants or live pups.*

## DISCUSSION

These results suggest no protective effect of the D2 uterine environment on ethanol teratogenesis. However there is some evidence to suggest the B6 uterine environment can increase susceptibility. But the lack of power due to genotype-dependent implantation failure limits the reliability of this finding. Vertebral malformations were increased in D2 fetuses transferred to B6 dams, but this result was not statistically significant because of too few viable D2 litters. *In utero* alcohol exposure produced similar malformation frequencies among B6 fetuses regardless of whether embryos were transferred into a B6 or D2 dam or they resulted from natural mating. This suggests ethanol exposure occurred during the same critical period of development among all B6 fetal groups. That is, the transfer procedure did not change a key developmental stage sensitive to digit, vertebral, and kidney malformations. Only fifteen percent of D2 blastocysts successfully implanted and only 35% of B6 blastocysts successfully implanted. A similar difference was observed in the percentage of viable pups produced when D2 or B6 blastocysts were implanted into pseudo-pregnant CByB6F1/J mice (Byers et al., 2006). Viable pup percentages were 25% for D2 implants but 53% for B6 implants. To assure meaningful comparisons on sample sizes, future studies should at least quadruple the number of litters with transferred D2 blastocysts and double the number of litters with transferred B6 blastocysts. Also, a focus on only skeletal (vertebral) malformations and not soft-tissue (kidney) malformations would increase sample size. A maternal effect for vertebral malformations and not kidney malformations was previously observed (Downing and Gilliam, 1999).

Susceptibility to specific ethanol teratogenic effects in mice appear to be due to both fetal and maternal genetic influences. By making assumptions about additive and dominance genetic effects, variation in total malformation frequency can be partitioned into what is due to embryonic genotype and what is due to maternal genotype. Interestingly, when maternal genetic influences are observed they account for more than half of the total malformation frequency (Gilliam and Irtenkauf, 1990; Gilliam et al., 1997, 2011; Downing and Gilliam, 1999). Recently, using stocks related to B6 and D2 mice, Lossie et al. (2014) identified specific genes showing up-regulation in B6 embryos but down-regulation in D2 embryos after ethanol exposure. These findings are consistent with others when using embryo cultures (Ogawa et al., 2005; Chen et al., 2011). Determining how these genes are differentially regulated by maternal factors would shed light on the maternal contribution to ethanol teratogenesis. Uncovering the reasons for a maternal effect in mice may lead to a better understanding of why only certain babies have FAS or FASD.

Because of the increased vertebral malformations observed in ethanol exposed D2 embryos transferred to B6 dams, it is tempting to speculate that the B6 uterine environment somehow changed fetal D2 gene expression. In support of this hypothesis, Downing et al. (2012) measured gene expression changes following *in utero* alcohol exposure in four embryonic genotypes: true-bred B6 and D2, and reciprocally bred B6D2 and D2B6. The mating period, time of intubation, and ethanol and maltose dosages were all nearly identical to those used in the present study. Of specific interest was the finding that 22 genes were differentially expressed in B6D2 embryos gestating within B6 dams but not in genetically identical D2B6 embryos gestating within D2 dams following *in utero* alcohol exposure. B6D2 fetuses also show significantly higher rates of ethanol teratogenesis than do D2B6 fetuses (Downing and Gilliam, 1999). These results implicate the B6 uterine environment as increasing FASD risk. These effects could also be due to changes in fetal and/or placental genomic imprinting status (Nelissen et al., 2011). The interplay between maternal genetic factors and fetal genetic factors that increase FASD susceptibility need further study to unravel the underlying mechanisms responsible.

## Conflict of Interest Statement

The author declares that the research was conducted in the absence of any commercial or financial relationships that could be construed as a potential conflict of interest.
